# Association of Systemic Sclerosis With Premature Ventricular Complexes and Cardiac Arrest: A National Inpatient Sample Analysis for 2021

**DOI:** 10.7759/cureus.84950

**Published:** 2025-05-28

**Authors:** Enyioma Nwogwugwu, Kayode E Ogunniyi, Ruth Pius, Godfrey Tabowei, Niyati Patel, Olufemi P Odugbemi, Oboseh Ogedegbe, Gabriel Alugba, Derek Ugwendum, Kenneth Ong

**Affiliations:** 1 Internal Medicine, Lincoln Medical and Mental Health Center, New York, USA; 2 Internal Medicine, Richmond University Medical Center, Staten Island, USA; 3 Department of Internal Medicine, Texas Tech University Health Sciences Center, Odessa, USA; 4 Internal Medicine, St George's University School of Medicine, True Blue, GRD; 5 Internal Medicine, Lincoln medical and mental health center, New York, USA; 6 Internal Medicine, Trinity Health Ann Harbor Hospital, Ypsilanti, USA; 7 Internal Medicine, Englewood Hospital and Medical Center, Englewood , USA; 8 Internal Medicine, Richmond University Medical Center Affiliated with Mount Sinai Health System and Icahn School of Medicine at Mount Sinai, New York, USA; 9 Cardiology, Lincoln Medical and mental health Center, New York, USA

**Keywords:** cardiac arrest, cardiac arrhythmias, cardiac scleroderma, myocardial fibrosis, premature ventricular complexes, scleroderma, scleroderma and cardiovascular disease, systemic sclerosis, ventricular arrhythmias, ventricular fib

## Abstract

Background: Systemic sclerosis (SSc) is a connective tissue disorder known to have multiple cardiovascular manifestations, including pulmonary fibrosis with pulmonary arterial hypertension, heart failure, and coronary artery disease. Arrhythmias, particularly ventricular arrhythmias, including premature ventricular complexes (PVC), ventricular tachycardia (VT), and ventricular fibrillation (VF), have been reported in patients with systemic sclerosis, but large-scale studies on their frequency and outcomes are limited.

Objective: We aimed to investigate whether SSc predisposes patients to ventricular arrhythmias and cardiac arrest, providing critical information for risk stratification and clinical management.

Methods: We analyzed discharges with a primary or secondary diagnosis of SSc (ICD-10 code M34). We compared the occurrence of PVC, VT, VF, and cardiac arrest between patients with SSc and those without. Univariate logistic regression was used to identify associations between SSc and arrhythmic events. Multivariate regression, adjusted for age, gender, smoking history, myocardial infarction, heart failure, hypertension, chronic kidney disease, and pulmonary fibrosis, was performed to assess independent associations. Statistical analyses were conducted using STATA 18.

Results: Among 28,729 discharges with a diagnosis of systemic sclerosis, univariate logistic regression showed significant associations between SSc and both PVC (OR 1.32; p=0.02) and cardiac arrest (OR 1.46; p<0.001). However, no significant associations were found for VT (p=0.5) and VF (p=0.6). After adjusting for confounders in multivariate logistic regression, PVC (OR 1.29; p=0.034) and cardiac arrest (OR 1.47; p<0.001) remained significantly associated with SSc, while VT (p=0.555) and VF (p=0.756) did not.

Conclusion: Patients with SSc experience a higher frequency of PVC and cardiac arrest compared to the general population, even after adjusting for comorbid risk factors. These findings highlight the importance of close cardiac monitoring in this patient population to enable early detection and management of arrhythmias. Future prospective studies are needed to further explore the role of fibrosis, long-term arrhythmia burden, and clinical outcomes in SSc.

## Introduction

Systemic sclerosis (SSc) is a chronic autoimmune disorder characterized by inflammation of tissue with multi-organ fibrosis, microangiopathy, and immune dysregulation. Although diverse, both primary and secondary cardiac involvement is increasingly understood as major contributors to morbidity and mortality. Cardiac involvement is often complex and requires multimodality imaging and biomarker evaluations. D’Angelo et al. studied 58 autopsy cases of SSc and demonstrated widespread organ fibrosis affecting the lungs, kidneys, gastrointestinal tract, skeletal muscle, myocardium, and pericardium [1]. Vascular pathology was also prominent, especially in the renal and pulmonary circulation, contributing to systemic and pulmonary hypertension. Patients with cardiac involvement, particularly those with metabolic myocardial fibrosis and pericardial disease, have very poor prognoses, with some reports suggesting up to 75% mortality in severe cardiac SSc involvement [2].

Cardiac manifestations can range from subclinical ventricular dysfunction to overt heart failure, pericarditis, coronary artery disease, and life-threatening arrhythmias. Despite their clinical importance, cardiac arrhythmias associated with SSc, particularly abnormal ventricular rhythms such as premature ventricular contractions (PVCs), ventricular tachycardia (VT), and ventricular fibrillation (VF), remain underrecognized. Previous studies, like the ones done by Fairley and colleagues, reported in a systematic review that the risk of PVCs in patients with SSc may be up to ten times higher than in healthy controls [3]. However, many of these studies were small or lacked nationally representative populations. Understanding the burden and predictors of these arrhythmias is crucial for risk stratification and guiding clinical management. Using a large national dataset, this study addresses the knowledge gap by examining the association between SSc and ventricular arrhythmias, including PVC, VT, VF, and cardiac arrest. Leveraging the scope of the National Inpatient Sample (NIS), we aim to evaluate whether SSc independently predisposes patients to ventricular arrhythmias and cardiac arrest beyond traditional cardiovascular risk factors and comorbidities.

Pathogenesis

Myocardial fibrosis is a hallmark of systemic sclerosis. It is believed to arise from chronic microvascular ischemia due to endothelial dysfunction, capillary rarefaction, and repetitive vasospastic episodes. These mechanisms cause ischemic reperfusion injury, leading to oxidative stress, fibroblast activation, and excessive collagen deposition within the myocardium. This remodeling is mediated by profibrotic cytokines such as transforming growth factor beta (TGF-β) and endothelin 1 [4]. Fibrosis disrupts both mechanical function and the cardiac conduction system, introducing regions of slowed conduction, reentry pathways, and heterogeneity in action potential propagation that increase susceptibility to arrhythmias. In addition, autonomic dysregulation due to fibrosis of cardiac ganglia and nerve fibers results in sympathetic predominance and reduced parasympathetic tone, factors known to heighten arrhythmic risk. These mechanisms have been corroborated by cardiac MRI studies, where late gadolinium enhancement (LGE) has been associated with ventricular arrhythmias in SSc [4,5].

Roberts et al. examined a prospective cohort of 50 patients with SSc and found that 32 percent exhibited conduction abnormalities (e.g., left anterior fascicular block, first-degree atrioventricular (AV) block), while 62% developed supraventricular or ventricular tachyarrhythmias6. Electrophysiological testing in this group revealed dysfunction across the sinus node, AV node, and intraventricular conduction system, findings consistent with fibrotic atrophy of conduction tissue. In the general population, PVC prevalence is typically 0.5 to 1%. However, our study found a rate of 1.2% among SSc patients, reinforcing the notion that SSc substantially increases arrhythmic burden, a finding consistent with prior studies [3,6]. Importantly, subtypes of the disease (e.g., diffuse vs. limited cutaneous SSc) were not identifiable in the data source, which limits stratified risk assessment based on clinical phenotype.

In addition to intrinsic myocardial disease, SSc is frequently associated with pulmonary arterial hypertension (PAH), which further exacerbates cardiac stress and arrhythmia risk. PAH in SSc is primarily caused by pulmonary vascular remodeling, vasoconstriction, and intimal fibrosis, often in the setting of chronic endothelial injury and inflammation, leading to elevated pulmonary artery pressures [7]. These changes contribute to increased right ventricular (RV) afterload, hypertrophy, ischemia, and maladaptive remodeling, thereby promoting both atrial and ventricular arrhythmias [7]. For instance, monomorphic VT may originate from scarred RV tissue. Atrial fibrillation is common in the setting of RV dysfunction, due to subsequent atrial dilation. Combined with autonomic imbalance and chronic pressure overload, these changes significantly raise the risk of PVCs and cardiac arrest. In SSc, PAH is often associated with a poor prognosis, as it can lead to RV failure as the heart struggles to overcome the increased afterload imposed by the pulmonary vasculature. Collectively, these mechanisms support the view that SSc patients are at heightened risk for arrhythmias and require intensive cardiac surveillance [7,8].

## Materials and methods

Data source

This study analyzes the 2021 NIS, which is publicly available along with other de-identified datasets curated by the Agency for Healthcare Research and Quality (AHRQ) within the Healthcare Cost and Utilization Project (HCUP). The NIS contains discharge data for short-term non-federal acute care hospitals located in the US, with a stringent sampling design using stratified probability sampling based on region, urban or rural classification, teaching status of the facility, ownership type, and hospital bed size. Within each stratum, about 20% of hospitals are randomly sampled, and discharge-level weights are applied to create estimates that are representative at the national level. After applying survey weights, the dataset represents approximately 28.4 million hospitalizations among adults in 2021. Because the data is fully de-identified and publicly available, the research did not require institutional review board approval or consent.

Study design and population

Using data from the 2021 NIS, this study undertakes a retrospective cross-sectional analysis of adult inpatient discharges. Patients aged 18 years and older were included. Discharges with SSc as a primary or secondary diagnosis were ascertained using the ICD-10-CM code “M34.” All other adult discharges not having this code were considered the non-systemic-sclerosis comparison group, resulting in approximately 28.37 million discharges. Patients with other autoimmune diseases, such as systemic lupus erythematosus or rheumatoid arthritis, were included, and the risk of bias arising from the coexisting autoimmune disease is noted in the limitations. Arrhythmic and cardiac arrest outcomes were extracted using ICD-10-CM diagnostic codes. Diagnosis of PVCs is documented with code I49.3, VT with I47.2 and I47.2x, VF with I49.01, and cardiac arrest is coded I46, I462, I468, and I469. Some of the covariates were also defined by ICD-10 codes and comprised age, sex, heart failure, prior myocardial infarction, diabetes mellitus, hypertension, chronic kidney disease, smoking, and interstitial lung disease. A complete list of codes can be found in the supplementary materials.

Outcomes

The primary outcomes of concern were the recorded cases of ventricular arrhythmias and cardiac arrest and their prevalence among patients who have SSc in comparison to patients without the condition. Specifically, we looked for the presence of PVCs, VT, VF, and cardiac arrest during the hospitalization. Given the NIS dataset’s constraints, there was no way to discern if and when, during the hospitalization, rhythmic disturbances took place. Also, we could not distinguish between periods of clinical observation and clinical arrhythmia, nor the clinical severity of the events.

Statistical analysis

The descriptive statistics were compiled to summarize baseline characteristics. Continuous variables were expressed as means plus standard deviations and tested by Student’s t-tests. Categorical variables were recorded as frequencies and proportions and tested by Pearson’s chi-square tests. Unadjusted odds ratios (ORs), 95% confidence intervals (CIs), and p-values were determined using univariate logistic regression to estimate the association between SSc and arrhythmias. Multivariable logistic regression models were developed to control for confounding factors. Adjusted models incorporated age, female sex, smoking, heart failure, history of myocardial infarction, diabetes mellitus, chronic hypertension, chronic kidney disease, and interstitial lung disease as covariates. These factors were chosen based on their known influence on the risk of developing arrhythmia and involvement in cardiovascular comorbidities related to systemic sclerosis. All statistical analyses were performed in Stata version 18 (StataCorp LLC, College Station, TX). National estimates and their variance were calculated using NIS-specific discharge-level weights and stratification variables with the survey (svy) command suite. These weights were applied in univariate and multivariate models to obtain nationally stratified representative estimates. A two-sided p-value less than 0.05 was considered significant.

Data limitations

The NIS dataset lacks information on the utilization of corticosteroids, immunosuppressive or antiarrhythmic medications, laboratory data, imaging studies including echocardiography or cardiac MRI, electrocardiograms, and left ventricular ejection fractions. The lack of outpatient and longitudinal follow-up data also hampers the evaluation of arrhythmia recurrence or progression. In addition, although ICD-10 codes are frequently employed in administrative and Literary studies, their NIS-specific validity for systemic scleroderma and associated arrhythmias has not been formally validated, raising the risk of misclassification. These limitations are notably discussed in the discussion section.

## Results

Patient characteristics

A total of 28,730 adult hospital discharges with a diagnosis of SSc were identified (Table 1). Patients with SSc were significantly older than those without (63.77 ± 14.56 vs. 58.00 ± 20.14 years, p < 0.0001). There was a marked female predominance in the SSc group compared to the non-SSc group (24,219 (84.3%) vs. ~15,907,772 (56.07%), p < 0.001).

**Table 1:** Characteristics of the study population

Variable	Systemic Sclerosis	No Systemic Sclerosis	ALL ADULT DISCHARGES	p-Value
Total	28730	28,371,270	28,400,000	
Age mean +- SD	63.76523+- 14.56	58.00438 +- 20.14	58.0102 +- 20.13242	<0.0001
Gender n (%) (<0.001)				
Male	4,510 (15.70%)	12,500,000 (43.93%)	12,500,000 (48.90%)	
Female	24,220 (84.30%)	15,900,000 (56.07%)	16,000,000 (56.10%)	
Hospital bed size n (%) (<0.001)				
Small	5,675 (19.75%)	6,573,785 (23.14%)	6,579,460 (23.13%)	
Medium	7,420 (25.83%)	7,899,642 (27.80%)	7,907,062 (27.80%)	
Large	15,635 (54.42%)	13,897,843 (49.06%)	13,913,478 (49.07%)	
Median Household Income for Patient’s ZIP Code (Socioeconomic Status) <0.001				
Quartile 1 n (%)	7,510 (26.47%)	8,504,959 (30.48%)	8,512,469 (30.47%)	
Quartile 2 n (%)	6,790 (23.94%)	7,109,612 (25.48%)	7,116,402 (25.48%)	
Quartile 3 n (%)	6,920 (24.40%)	6,644,288 (23.81%)	6,651,208 (23.81%)	
Quartile 4 n (%)	7,145 (25.19%)	5,646,380 (20.23%)	5,653,525 (20.24%)	
Race (0.001)				
Caucasian n (%)	18,925 (67.36%)	18,100,000 (65.42%)	18,100,000 (65.42%)	
Black/African American n (%)	4,110 (14.63%)	4,328,629 (15.65%)	4,332,739 (15.65%)	
Hispanic n (%)	3,520 (12.53%)	3,430,596 (12.40%)	3,434,116 (12.40%)	
Asian or Pacific Islander n (%)	705 (2.51%)	800,204 (2.89%)	800,909 (2.90%)	
Native American n (%)	220 (0.78%)	197,455 (0.71%)	197,675 (0.71%)	
Others n (%)	615 (2.19%)	808,380 (2.92%)	808,994 (2.92%)	
Smoking Status (codes Z720, Z87891, and code stem F17)	9,000 (31.33%)	10,200,000 (35.78%)	10,200,000 (35.78%)	<0.001
Comorbidities				
Previous myocardial infarction n (%)	1,135 (3.95%)	780,254 (2.75%)	781,389 (2.75%)	<0.001
Heart failure n (%)	9,050 (31.50%)	5,253,648 (18.49%)	5,262,698 (18.50%)	<0.001
Hypertension n (%)	8,750 (30.45%)	8,780,988 (30.90%)	8,789,738 (30.90%)	0.463
Chronic kidney disease n (%)	6,770 (23.56%)	5,196,945 (18.29%)	5,203,715 (18.30%)	<0.001
Interstitial lung disease n (%)	5,660 (19.70%)	260,580 (0.92%)	266,240 (0.94%)	<0.001

Hospital bed size distribution differed between groups, with SSc patients more frequently admitted to large-bed hospitals (15,634 (54.42%) vs ~13,918,946 (49.06%), p <0.001). Socioeconomic status, determined by median household income quartiles, showed that a lower proportion of SSc patients resided in the lowest income quartile compared to those without SSc (7,605 (26.47%) vs. 8,647,563 (30.48%), p < 0.001).

The racial composition was broadly similar, although a slightly higher proportion of patients with SSc identified as Caucasian (19,352 (67.36%) vs.18,560,485 (65.42%), p = 0.001). Smoking status was less frequent in the SSc group (9,001 (31.33%) vs. ~10,151,241 (35.78%), p < 0.001).

Regarding comorbidities, SSc patients showed a higher prevalence of previous myocardial infarction (1,135 (3.95%) vs. 780,210 (2.75%), p < 0.001), heart failure (9,050 (31.50%) vs. ~5,245,848 (18.49%), p < 0.001), and chronic kidney disease (6,769 (23.56%) vs. 5,189,105 (18.29%), p < 0.001). While the prevalence of hypertension was similar between groups (8,748 (30.45%) vs. ~8,766,723 (30.90%), p = 0.463), interstitial lung disease was substantially more common among those with SSc (5,660 (19.70%) vs. ~261,016 (0.92%), p < 0.001).

Unadjusted clinical events

Clinical event rates among patients with SSc are compared with patients without the condition in Table 2. In unadjusted analyses, PVCs were reported in 344 out of 28,729 SSc patients (1.20%) versus approximately 257,175 out of 28,371,271 non-SSc patients (0.91%) (unadjusted OR 1.32; p=0.02). Cardiac arrest was also more frequent among patients with SSc, 451 out of 28,729 (1.57%) versus approximately 306,412 out of 28,371,271 non-SSc patients (1.08%) (unadjusted OR 1.46; p<0.001). Conversely, there were no significant unadjusted differences in the rates of VF or VT, and percutaneous coronary intervention (PCI) rates did not differ significantly (OR 0.83, p = 0.173). However, in-hospital mortality was notably higher in the SSc cohort, noted in 1,486 out of 28,729 patients (5.17%) vs. approximately 991,247 out of 28,371,271 non-SSc patients (3.51%) (unadjusted OR 1.50; p<0.001).

**Table 2:** Clinical events

Event Prevalence	Systemic Sclerosis	No Systemic Sclerosis	Odds Ratio or Difference	p-value
Premature ventricular complexes n (%)	344 (1.20%)	258530 (0.91%)	1.32	0.020
Ventricular tachycardia n (%)	540 (1.89%)	498435 (1.75%)	1.07	0.504
Ventricular fibrillation n (%)	75 (0.26%)	85565 (0.30%)	0.87	0.601
Cardiac arrest n (%)	450 (1.57%)	306630 (1.08%)	1.46	<0.001
Cardioversion n (%)	255 (0.89%)	210460 (0.74%)	1.20	0.221
Percutaneous coronary intervention rates n (%)	265 (0.92%)	314200 (1.11%)	0.83	0.173
In-hospital mortality n (%)	1485 (5.17%)	998413 (3.51%)	1.497	<0.001

Adjusted clinical outcomes

After controlling for age, gender, pre-existing heart failure, previous myocardial infarction, diabetes mellitus, hypertension, chronic kidney disease, smoking status, and interstitial lung disease (Table 3), several key findings emerged. In the fully adjusted model, the association between SSc and PVCs remained significant (adjusted OR 1.28, 95% CI 1.01-1.62, p=0.043), although the effect size was slightly attenuated compared to the unadjusted estimate. Patients with SSc continued to have a higher likelihood of cardiac arrest (adjusted OR 1.42, 95% CI 1.15-1.76, p=0.001), and in-hospital mortality remained significantly elevated (adjusted OR 1.23, 95% CI 1.09-1.40, p=0.001). In contrast, there were no significant adjusted associations between SSc and ventricular tachycardia, ventricular fibrillation, cardioversion, or PCI (p>0.05 for all comparisons). The unadjusted and adjusted odds ratios, CIs, and p-values are summarized in Table 3.

**Table 3:** The unadjusted and adjusted odds ratios, CIs, and p-values

Event Prevalence						
	Unadjusted OR/Difference	Confidence Interval	p-value	Adjusted OR/Difference	Confidence Interval	p-value
Premature ventricular complexes (%)	1.32	1.05 - 1.68	0.020	1.28	1.007968 1.622437	0.043
Ventricular tachycardia (%)	1.07	0.87 - 1.32	0.504	1.06	0.86 - 1.31	0.561
Ventricular fibrillation	0.60	0.51 - 1.48	0.601	0.95	0.56 - 1.64	0.864
Cardiac arrest	1.46	1.19 - 1.79	<0.001	1.42	1.15 - 1.76	0.001
Cardioversion	1.20	0.896 - 1.608	0.221	1.08	0.80 - 1.45	0.624
Percutaneous coronary intervention rates	0.83	0.64 - 1.08	0.173	1.13	0.87 - 1.48	0.359
In-hospital mortality	1.497	1.33- 1.69	<0.001	1.23	1.09 - 1.395	0.001

Ischemia is a major contributor to ventricular arrhythmias, so differences in PCI were evaluated as a proxy for clinically significant myocardial ischemia. In both unadjusted and adjusted analyses, PCI rates did not differ significantly between the two groups (adjusted OR 1.13, 95% CI 0.87-1.48, p = 0.359).

Overall, these findings indicate that patients with SSc are at increased risk of certain cardiac complications, including premature ventricular complexes, cardiac arrest, and higher in-hospital mortality, even after adjusting for key risk factors and comorbid conditions.

## Discussion

In this study, we investigated the association between SSc diagnosis and ventricular arrhythmias, including PVC, VT, and VF, and cardiac arrest among hospitalized patients diagnosed with SSc in 2021. For admitted patients with a SSc diagnosis compared to those without, we observed a significantly higher likelihood of having PVCs, having a cardiac arrest, and experiencing in-hospital mortality. The strength of these associations remained significant after controlling for other comorbid illnesses and demographic variables, suggesting they may be causal.

These findings align with earlier studies associating SSc with higher cardiovascular complications and mortality. For example, cardiac involvement in SSc has been shown to contribute significantly to mortality in the disease [3]. Although cardiac involvement in SSc can include the endocardium, pericardium, and myocardium, myocardial involvement is most strongly associated with an increased risk of arrhythmia [9-11], which further contributes to poor outcomes [12]. As mentioned earlier, Fairley et al. [3] found that SSc patients were found to be almost ten times more likely to suffer from frequent PVCs than their healthy counterparts. Although our study reported a smaller effect size (OR 1.3), it is likely due to the comparator groups used. In our study, the control group also comprises hospitalized patients with an overall increased incidence of PVCs compared to the non-hospitalized, general healthy population. Also, PVCs have been associated with poor prognosis in patients with structural heart disease [12], which represents a significant proportion of SSc patients [13]. By extension, one may infer that the occurrence of PVCs in SSc patients is associated with poor outcomes.

Even though population-based studies indicate that SSc is linked with an increased risk of sudden cardiac death, the specific association between SSc and cardiac arrest during a patient’s hospital stay has not been well explored. This study finds that patients with SSc have a 50 percent greater likelihood of having a cardiac arrest compared to non-SSc counterparts, after adjusting for conventional cardiovascular risk factors. Likewise, there was a 25% increased likelihood of in-hospital mortality in SSc patients compared to other hospitalized patients, irrespective of the reason for admission. In-hospital mortality among SSc patients has been reported to show a downward trend in the past few decades. Goel et al. showed in their 20-year analysis of hospitalized scleroderma patients in the US that in-hospital mortality had decreased from 9.63% in 1993 to 8.4% in 2013 [14]. In line with this, our study shows a further decrease in in-hospital mortality to 5.17%. This downward trend may be attributed to increased physician awareness and advancement in treatment modalities for SSc complications. Interestingly, it was shown by Yen and colleagues that the decrease in mortality of SSc patients is more pronounced among patients who are below the age of 65 years [15].

An important consideration in our analysis is the potential underestimation of ventricular arrhythmias when evaluating VT and VF as separate outcomes. VT and VF often lead to cardiac arrest, and in many instances, clinical coding may prioritize the broader diagnosis of cardiac arrest rather than specifying the underlying arrhythmic event. This could explain why VT and VF alone did not reach statistical significance, while cardiac arrest remained significantly associated with SSc. The overlap between these conditions suggests that cardiac arrest may serve as a more inclusive marker of severe ventricular arrhythmias in this population. However, given the limitations of administrative coding, we cannot determine the exact proportion of cardiac arrest cases that were primarily driven by VT or VF versus other etiologies such as asystole or pulseless electrical activity.

Irrespective of the overall decrease in mortality of hospitalized patients with SSc, such patients still have an increased likelihood of inpatient death compared to those without SSc, even when controlled for comorbidity confounders and admission status. Potera et al. attributed this heightened risk of in-hospital death among SSc patients to their predisposition to infections and cardiovascular pathologies [16]. Further research should include composite arrhythmic endpoints and leverage rich clinical data from telemetry and cardiac imaging for more refined event detection and risk assessment in this population.

Clinical implications

Despite controlling for comorbidities, SSc patients are more likely to experience cardiac arrest and ventricular arrhythmias, especially PVCs. Physicians should closely monitor these individuals. Early intervention and the avoidance of life-threatening situations depend on routine screening for arrhythmias using Holter monitoring and ECGs. To improve patient outcomes, particularly for patients with other cardiovascular diseases, tailored management based on arrhythmic risk should be implemented. Management based on risk stratification might include beta-blocker therapy, electrophysiologic evaluation, and implantation of cardioverter defibrillators in select high-risk patients. A multidisciplinary approach involving cardiologists and rheumatologists is needed to provide comprehensive care to SSc patients.

Research implications

Subsequent investigations need to address the gaps left by prior studies regarding SSc and its potential arrhythmogenic mechanisms. More research is required to understand the contribution of myocardial fibrosis, microvascular disease, and imbalance of the autonomic nervous system to the development of persistent arrhythmias. There is a need to investigate the impact of arrhythmias on long-term survival, quality of life, and functional status in SSc patients. This population also needs to be the focus of clinical trials evaluating the effectiveness of antiarrhythmic and device-based therapies. Expanding the scope of risk assessment and defining tailored prevention strategies can be achieved by integrating advanced imaging techniques such as cardiac MRI, cardiac biomarkers, and multidimensional clinical profiling.

Strengths and limitations of the study

This study’s most significant strength is its use of a large, nationally representative inpatient dataset, which increases generalizability and enhances statistical power. Multivariable logistic regression allowed for the adjustment of a significant number of confounding variables such as age, gender, smoking status, previous cardiovascular disease, and pulmonary fibrosis. Such methodologies support the credibility of the covariate-adjusted associations between SSc and the heightened risk of PVCs, cardiac arrest, and inpatient mortality.

These findings are informative; however, important limitations must be acknowledged. The study’s retrospective design introduces potential selection bias and limits causal inference. The reliance on administrative coding may have led to underreporting of some arrhythmias, particularly those that were asymptomatic or transient. The lack of data on medications, outpatient surveillance, echocardiographic assessments, and cardiac MRI restricted the ability to explore the underlying mechanisms in detail. Furthermore, the analysis was limited to the hospitalization period and did not capture long-term outcomes after discharge. These limitations highlight the need for future prospective studies to investigate the clinical course of SSc and its arrhythmic complications in greater depth.

## Conclusions

This study demonstrates that SSc is independently associated with an increased risk of PVC and cardiac arrest, even after adjusting for key cardiovascular risk factors. While VT and VF alone did not reach statistical significance, the significant association with cardiac arrest suggests that SSc patients may have a higher burden of life-threatening arrhythmias than previously recognized. These findings highlight the need for heightened cardiac monitoring and risk stratification in this population to facilitate early detection and management of arrhythmias. Future studies should focus on refining arrhythmia classification, exploring the underlying pathophysiological mechanisms, and evaluating targeted interventions to improve outcomes in SSc patients.
